# Supplementary figures and images for: Genetic Analyses of Response of Local Ghanaian Tanzanian Chicken Ecotypes to a Natural Challenge with Velogenic Newcastle Disease Virus
Source: Animals (Basel). 2022 Oct 13;12(20):2755. doi: 10.3390/ani12202755 (PMC9597780; doi:10.3390/ani12202755)

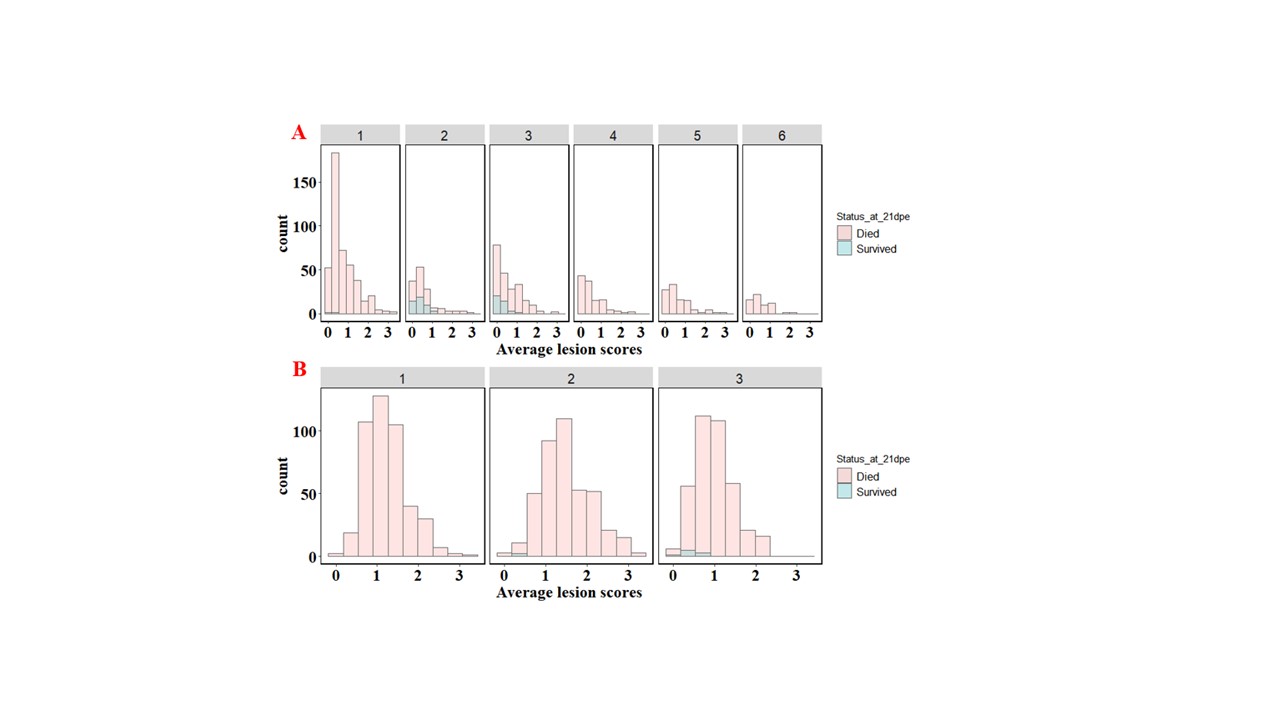

Supplement: Supplementary file 1 [file animals-12-02755-s001.zip › animals-1879645-supplementary.jpg]
